# Size matters: the biochemical logic of ligand type in endocrine crosstalk

**DOI:** 10.1093/lifemeta/load048

**Published:** 2023-12-08

**Authors:** Jameel Barkat Lone, Jonathan Z Long, Katrin J Svensson

**Affiliations:** Department of Pathology, Stanford University School of Medicine, Stanford, CA 94305, United States; Department of Pathology, Stanford University School of Medicine, Stanford, CA 94305, United States; Department of Chemistry, Stanford University, Stanford, CA 94305, United States; Stanford Diabetes Research Center, Stanford University School of Medicine, Stanford, CA 94305, United States; Sarafan ChEM-H, Stanford University, Stanford, CA 94305, United States; Wu Tsai Human Performance Alliance, Stanford University, Stanford, CA 94305, United States; Department of Pathology, Stanford University School of Medicine, Stanford, CA 94305, United States; Stanford Diabetes Research Center, Stanford University School of Medicine, Stanford, CA 94305, United States; Sarafan ChEM-H, Stanford University, Stanford, CA 94305, United States; Wu Tsai Human Performance Alliance, Stanford University, Stanford, CA 94305, United States; Stanford Cardiovascular Institute, Stanford University School of Medicine, CA 94305, United States

**Keywords:** endocrine, ligand size, metabolites, signaling, evolution

## Abstract

The endocrine system is a fundamental type of long-range cell–cell communication that is important for maintaining metabolism, physiology, and other aspects of organismal homeostasis. Endocrine signaling is mediated by diverse blood-borne ligands, also called hormones, including metabolites, lipids, steroids, peptides, and proteins. The size and structure of these hormones are fine-tuned to make them bioactive, responsive, and adaptable to meet the demands of changing environments. Why has nature selected such diverse ligand types to mediate communication in the endocrine system? What is the chemical, signaling, or physiologic logic of these ligands? What fundamental principles from our knowledge of endocrine communication can be applied as we continue as a field to uncover additional new circulating molecules that are claimed to mediate long-range cell and tissue crosstalk? This review provides a framework based on the biochemical logic behind this crosstalk with respect to their chemistry, temporal regulation in physiology, specificity, signaling actions, and evolutionary development.

## Background

### The endocrine system

The endocrine system is a large network of organs in the body that produces, stores, and secretes blood-borne factors called hormones. These hormones serve as the central long-range communication system of the body and maintain various physiological states. The discovery of endocrine organs and endocrine hormones dates back to the observation that the removal of specific organs would induce dramatic phenotypes, including disease or even death. Remarkably, these phenotypes could be entirely reversed by re-administration of crude biochemical preparations of the organs that were excised. These observations gave rise to the hypothesis that specific “factors” secreted by these excised organs were important for normal homeostasis and health. However, within the endocrine system, the roles of molecules traditionally labeled as “signaling metabolites” or “hormones” are not strictly defined, as they share many similarities. Distinctive features, such as their contribution to metabolic reactions—where metabolites often serve as precursors or intermediates and hormones exert regulatory control—provide a basis for differential consideration. Additionally, the specificity and affinity of receptor interactions offer further delineation; metabolites generally exhibit lower affinity and broader receptor interactions, contrasting with the high-affinity, targeted receptor binding characteristic of hormones. However, contextual factors may confer attributes of both systems to each class. The eventual biochemical purification and identification of such factors, which include insulin from the pancreas [1, 2], thyroid hormone from the thyroid gland [3–5], glucocorticoids from the adrenal gland [6], and sex hormones from the reproductive organs [7], provided direct evidence of the presence of endocrine hormones, and by extension, endocrine communication.

In addition to the traditional biochemical, activity-guided fractionation methods, newer genetic techniques have also brought forth a new era of endocrine hormone discovery and research into endocrine communication. For instance, hormones such as fibroblast growth factor 21 (FGF21) [8, 9], leptin [10], and growth differentiation factor 15 (GDF15) [11–14] were identified, not from organ resections, but through genetic screening and characterization. Interestingly, unlike classical endocrine organs like the thyroid or pancreas, the cell types and organs or tissues producing these hormones cannot be easily resected or even clearly delineated. This highlights the increasing complexity of the origins of endocrine hormones, with many tissues contributing to organ communication beyond classical glands alone.

### Why does the body rely on endocrine signaling?

French physiologist Claude Bernard was the first to suggest that the exchange of chemical messengers is essential for maintaining the stability of the internal environment, or “milieu interieur” [15]. Signaling involves a complex series of interactions beginning with the release of a secreted signaling molecule or ligand. The ligand travels by blood to interact with a specific receptor on a target cell. This interaction triggers a cascade of intracellular events, often mediated by second messengers, leading ultimately to a specific cellular response. The response might be a change in gene expression, protein function, or cell behavior [16, 17]. The endocrine system serves to integrate multiple physiological processes, ensuring that organs and tissues function in a coordinated manner, especially in response to external or internal challenges.

Disruptions of endocrine communication can result in various disorders. The translation of endocrine hormones to new therapeutics has dramatically improved human health. Most classically, deficiency of insulin from the pancreas results in diabetes, leading to persistently elevated blood glucose levels due to impaired glucose uptake in peripheral tissues, particularly in muscle and adipose tissues [18, 19]. The absence of insulin is central to diabetes, a condition that was once invariably lethal. The discovery that diabetic symptoms could be managed, or even reversed, by injecting insulin paved the way for endocrine research [2]. Abnormalities in adrenal, parathyroid, and reproductive function can lead to disorders such as adrenal disorders, parathyroid disorders, reproductive disorders, and tumors. Another example is leptin deficiency, which leads to abnormal energy metabolism, hyperphagia, and obesity [10]. Likewise, defects in growth hormone (GH) or insulin-like growth factor can lead to dwarfism. Therapeutically, synthetic GH addresses growth deficiencies in children and adults, while leptin therapy can normalize body weight in cases of congenital leptin deficiency.

### Recent advances and the need for a new framework

Over the past few decades, the development of more sensitive mass spectrometry methods, including proteomics, peptidomics, lipidomics, and metabolomics [20, 21], in combination with genetic approaches has accelerated the discovery of signaling molecules. The success of such techniques in screening novel molecules is exemplified by the fact that novel adipokines such as isthmin-1 (ISM-1) and ependymin-related protein 1 (EPDR1) have been discovered with the aid of proteomics-based mapping of secretomes [22, 23]. In parallel, N glucosyl taurine and supra basin-derived peptides have been discovered by metabolomics and peptidomics, respectively [24, 25]. This development has enabled an expanded understanding of intercellular communication. However, since each new molecule brings a unique aspect of biology, our current classification and frameworks primarily based on organ expression might not fully encompass the physiological relevance, regulation, and function of these signaling molecules. While our present knowledge has provided a solid foundation, it is helpful to have a comprehensive framework that also incorporates an understanding of their differences in production, their modes of action, their targets, and their regulatory processes. Moving forward, the line among traditional hormones, cytokines, and other signaling molecules may blur, broadening our understanding of how cells communicate over long distance. Here, we discuss a structured way to study these molecules, helping to understand their roles in health and disease.

## Functions, specificity, and selectivity of cellular targets

**Figure 1:**
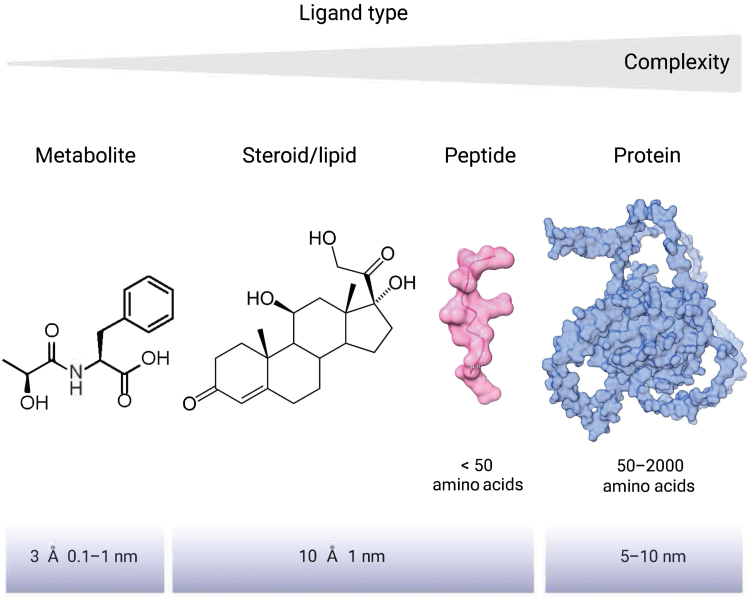
Structures and complexities of selected ligand types. The figure illustrates a spectrum of molecular complexities and sizes, displaying from right to left. On the far right, the structure of the secreted protein ISM-1 is shown, representing the larger and more complex end of the spectrum. Moving leftward, the structure of an 11-mer peptide is depicted, which is smaller and less complex than proteins. Further left, the structure of cortisol represents a middle-of-the-range molecule in terms of size and complexity. On the far left, the structure of Lac-Phe is presented, highlighting a relatively simple metabolite. The diversity in size, complexity, and formation of each structure underscores the sophisticated nature of intercellular and inter-organ communication.

Small molecules (metabolites, lipids), peptides, and proteins are all essential for various biological processes and cellular signaling, but they exhibit differences in their structure, size, function, and mechanisms of action (Fig. 1). Secreted proteins have diverse functions, including enzyme, signaling, and regulation of gene expression. They are large, often complex molecules composed of several chains of amino acids and have a molecular weight that can range from a few thousand to several million daltons (Da). Given that proteins have unique three-dimensional structures that determine their function and interactions with other molecules, they generally act through specific protein-protein interactions, which depend on the structural complementarity between the interacting partners [26]. These interactions can be transient or stable, depending on the nature of the proteins involved and the cellular context. Peptides (< 50 amino acids in size), small molecules, and lipids, on the other hand, are low-molecular weight compounds. Small molecules typically have a molecular weight of less than 900 Da [27]. They consist of a limited number of atoms and are composed of relatively simple structures, such as sugars, lipids, and amino acids. Secreted small molecules play a wide range of roles in biological processes, including energy metabolism, cellular signaling, and serving as building blocks. They can act as neurotransmitters, hormones, or secondary messengers in cell signaling pathways (Fig. 1).

### Selectivity and specificity

There are several reasons why nature utilizes different chemical modalities for cell and tissue crosstalk. The selection of ligands, determined by their size and other chemical properties, dictates the tissue-specific expression and functionality of proteins and small molecules. This diversity allows for a high degree of specificity and selectivity in interactions between signaling molecules and their target receptors [28]. With this large collection of molecular sizes and structures available, cells can fine-tune their interactions, ensuring that each signaling molecule can selectively activate or inhibit its target without disrupting unrelated pathways. The minimal specificity characteristic of the receptors for these ligands allows for specific interactions, which is a cornerstone of pharmacological interventions [29]. While small molecules easily diffuse through extracellular spaces to reach their target cells, larger peptides and proteins often require specific transport mechanisms such as receptor-mediated internalization, vesicle-mediated transport, or carrier proteins. Small molecules and lipids can often diffuse through cell membranes and extracellular spaces, allowing for rapid and direct interactions with target molecules or receptors. Smaller signaling molecules frequently bind to protein targets via non-covalent interactions, such as hydrogen bonding, ionic interactions, or hydrophobic interactions. For instance, FGF21, FGF19, and leptin are proteins that act as hormones and signal through specific receptors. FGF21 and FGF19 act via the FGF receptors (FGFRs) 1c, 2c, and 3c in conjunction with the obligate co-receptor β-Klotho [9, 30]. FGFRs 1, 2, and 3 are predominantly expressed in white adipose, brown adipose, and brain tissues. Interestingly, the activity of FGF19 for FGFR1c/β-Klotho is regulated by a single amino acid in the C-terminus of FGF19 [31]. β-Klotho, a shared co-receptor of FGF19 and FGF21, mediates its pharmacological functions in tissue-specific manner. For example, β-Klotho in the liver and adipocytes is dispensable for the effects of FGF19 and FGF21 on weight loss [32]. However, it is indispensable in neurons, where FGF19, FGF21, and bFKB1 (bispecific FGFR1/β-Klotho-activating antibody) require the β-Klotho receptor complex to exert their weight loss functions.

Similarly, leptin is a highly selective signaling molecule, binding to the leptin receptor (LEPR) with high specificity [33]. The structural features of leptin, including its four-helix bundle configuration, allow it to interact only with LEPR. Glucagon-like peptide-1 (GLP-1) and neuropeptide Y (NPY) are peptide hormones, and they also signal through specific receptors, commonly G-protein coupled receptors (GPCR). GLP-1’s selectivity is due to its unique peptide sequence fitting into the binding pocket of GLP-1 receptor (GLP-1R). NPY signals via multiple GPCRs (Y1, Y2, Y4, Y5), showing selectivity based on sequence variations between different NPY family peptides [34]. On the other hand, prostaglandins (PGs) are lipid-based signaling molecules that interact with a series of GPCRs (PGD2 receptor (DP), PGE2 receptors (EP1 − 4), PGF2α receptor (FP), PGI2 receptor (IP), thromboxane A2 receptor (TP), chemoattractant receptor-homologous molecule expressed on T_H_2 cells (CRTH2)). The diversity of receptors allows for high selectivity based on the specific structure of each PG. Fatty acid esters of hydroxy fatty acids (FAHFAs), and palmitic acid esters of hydroxy stearic acids (PAHSAs) are specific types of lipids that have been identified as signaling molecules involved in various metabolic processes, including insulin sensitivity, insulin secretion, and thermogenesis [35, 36]. FAHFA/PAHSA lipids signal via multiple receptors, including GPCRs and nuclear receptors. The specificity of these interactions depends on the chain length and degree of saturation of fatty acids. Lastly, lactate and serotonin, while both being metabolites, differ significantly in their signaling mechanisms. As a signaling molecule, lactate exhibits low specificity and interacts with a broad spectrum of protein receptors, G protein-coupled receptor 81 (GPR81, also known as hydroxycarboxylic acid receptor 1 (HCA1)) in certain tissues. Its signaling is less specific than the proteins and peptides described above, likely due to its simpler molecular structure. Serotonin (5-hydroxytryptamine) is a biogenic amine that signals via an array of GPCRs and ligand-gated ion channels, making it a highly specific and versatile signaling molecule [37, 38].

To understand the development of specificity, it is helpful to elucidate its origin from an evolutionary perspective. The synthesis of signaling molecules requires a certain metabolic state and a considerable amount of energy. Thus, cells have evolved mechanisms to select signal molecules that are metabolically economical and space saving. It is interesting to observe a conserved uniformity among organisms in employing smaller signaling molecules for quick and frequent responses like neuronal transmission and muscle contraction [39]. Why does an organism recruit different sizes of signal molecules? The answer might lie in versatility contributed by the availability of different signal molecules where size plays an important role. For example, neurotransmitters like dopamine and γ-aminobutyric acid (GABA) can diffuse in less than 1 millisecond across 20 nm synaptic cleft [40]. Smaller molecules confer a great advantage to an organism in mediating a rapid signaling event because smaller molecules are mobile and can be stored in higher amounts as a readily accessible signal pool. Moreover, the synthesis of signaling molecules demands a particular metabolic stage and requires a large amount of energy [41].

In summary, the nature of the ligand (protein, peptide, lipid, metabolite) greatly influences its receptor specificity and signaling selectivity (Table 1). These characteristics, in turn, reflect the complexity of the molecular structures and the specific needs of the tissues in which they function.

**Table 1.** Ligand size of different classes of secreted signaling molecules and their established roles in metabolism.

Class	Molecule	Ligand size	Source	Known role in metabolism	References
Metabolites	Acetylcholine	0.5 Å	Neurons	Stimulates muscle contraction, involved in learning and memory	[42, 43]
	Serotonin	0.5 Å	Brain, gut	Regulates mood, appetite, and sleep, also affects memory and learning	[37, 38]
	Norepinephrine	0.5 Å	Brain, sympathetic nervous system	Affects attention and response actions, involved in the fight-or-flight response	[44, 45]
	Lactate	0.5 Å	Muscles, red blood cells, brain, other tissues	Waste product of anaerobic metabolism, also used as an energy source by other tissues	[46, 47]
	Succinate	0.5 Å	Mitochondria	Involved in the citric acid cycle, signal hypoxia and inflammation	[48]
	β-hydroxybutyrate	0.5 Å	Liver	Used as an energy source during periods of fasting or intense exercise	[49]
	Lac-Phe	1 Å	Macrophages	Secreted after exercise, known to suppress appetite	[50]
	T4	1 nm	Thyroid gland	Regulates metabolism and growth, controls rate of energy use	[3, 51]
	Kynurenine	1 Å	Liver, other tissues	Plays a role in the regulation of immune responses and neuroactive signaling	[52]
	Acylcarnitines	1.5 Å	Various tissues	Involved in fatty acid metabolism, transport fatty acids into mitochondria for β-oxidation	[53, 54]
Steroids/lipids	Resolvins/maresins	0.5 nm	White blood cells, certain tissues	Involved in the resolution of inflammation, promote tissue regeneration	[55, 56]
	Prostaglandins	1.5 nm	Various cells	Regulate inflammation, involved in the regulation of bone metabolism	[57, 58]
	Sphingolipids	1.5 nm	Various cells	Regulate cellular processes including differentiation, proliferation, cell signaling, and apoptosis	[59, 60]
	Cholesterol	1 nm	Liver, dietary intake	Function as structural molecule in cell membranes and precursor for steroid hormones, regulates SREBP transport	[61, 62]
	FAHFA	1.5 nm	Various tissues	Modulate insulin sensitivity, inflammation, and thermogenesis	[35]
	PAHSA	1.5 nm	Various tissues	Potent regulators of glucose homeostasis and insulin secretion	[36]
	Testosterone	1.5 nm	Testes, adrenal glands	Promotes muscle and bone growth, stimulates production of red blood cells	[63]
	Estrogen	1.5 nm	Ovaries, adrenal glands	Plays a role in energy balance and glucose homeostasis	[7, 64]
	Glucocorticoids (Cortisol)	2 nm	Adrenal gland	Regulates metabolism and immune response, helps body respond to stress	[6, 65]
Peptides	GLP-1	30 AA	Intestine	Enhances insulin secretion and reduces glucagon secretion	[66, 67]
	Ghrelin	28 AA	Stomach	Stimulates appetite and food intake, also plays a role in energy balance	[68]
	NPY	36 AA	Brain, nervous system	Potent stimulator of food intake, influences energy homeostasis	[34, 69]
	CCK	58 AA	Intestine	Stimulates digestion of fat and protein through the release of digestive enzymes from the pancreas	[70, 71]
	Oxytocin	9 AA	Hypothalamus	Stimulates contraction of uterus and milk ejection in breastfeeding, promotes bonding and social behavior	[72, 73]
	Secretin	27 AA	S cells of the small intestine	Regulates water homeostasis and secretion of gastric juice	[74, 75]
	Amylin	37 AA	Beta cells of the pancreas	Slows gastric emptying, promotes satiety	[76, 77]
	PYY	36 AA	L cells in the ileum and colon	Reduces appetite, slows gastric emptying	[78]
Proteins	Glucagon	3.5 kDa	Pancreas	Stimulates conversion of stored glucose (glycogen) in the liver into glucose	[79, 80]
	Insulin	5.8 kDa	Pancreas	Regulates glucose metabolism, promotes the storage of glucose	[1, 81]
	Leptin	16 kDa	Adipose tissue	Regulates appetite and energy expenditure	[10]
	FGF21	22.3 kDa	Liver	Regulates glucose and lipid metabolism	[9, 82]
	FGF19	21.8 kDa	Ileum	Regulates bile acid synthesis and energy homeostasis	[83, 84]
	GDF15	35 kDa	Multiple	Suppresses appetite, enhances glucose and lipid metabolism	[11–14]
	NRG4	12 kDa	Liver, brown adipose tissue	Regulates hepatic lipogenesis and systemic energy metabolism	[85]
	Adiponectin	28 kDa	Adipose tissue	Regulates glucose levels and fatty acid breakdown	[86]
	ISM-1	52 kDa	Adipose tissue, various tissues	Regulates glucose uptake and lipid synthesis	[23]
	PM20D1	55 kDa	Various tissues	Involved in the biosynthesis of N-acyl amino acids and energy expenditure	[87]
	Dkk3	38	Skeletal muscle	Involved in muscle differentiation and regeneration	[88]
	Apo-B48	240 kDa	Intestine	Involved in transport of dietary lipids from intestines to peripheral tissues (primarily adipose and skeletal muscle)	[89]
	Apo-B100	550 kDa	Liver	Involved in liver-mediated removal and metabolism of LDL	[90]

Å, angstrom; nm, nano meter; kDa, kilo dalton.

### Modulation of signaling strength and duration: spatial, temporal, and chemical modalities

Key elements such as the spatial distribution, temporal aspects, and chemical nature of signals determine the routing of information and its physiological impact. The spatial distribution, timing, and chemical nature of signals can change both the intensity and durability, which is fundamental for managing complex cellular activities. Cells employ different modes of signaling to adapt to these temporal variations: paracrine for short distances and endocrine for longer distances. Both the size and structure of the signaling molecules contribute to the versatility and specificity of cellular responses, with smaller molecules being advantageous in mediating rapid signaling events due to their high mobility and greater storage capacity. For instance, the molecular size of endocrine peptides, such as triiodothyronine (T3), thyrotropin-releasing hormone (TRH), and met-enkephalin, correlates inversely with their signaling speed, enabling rapid diffusion and receptor engagement. Small peptides likely can quickly traverse biological fluids and initiate fast physiological responses due to reduced steric hindrance and high receptor affinity. In terms of time scale, small molecules typically work faster as they are often under enzymatic regulation, providing rapid control of production and degradation. Larger molecules are often subject to transcriptional regulation and usually respond at a slower pace. Small molecules such as neurotransmitters (e.g. dopamine, serotonin, and acetylcholine) diffuse rapidly, leading to rapid and frequent responses such as neuronal transmission and muscle contraction. On the other hand, larger molecules, including proteins (e.g. insulin, glucagon, and growth factors) and larger peptides, exhibit slower diffusion rates, allowing for sustained signaling. These larger molecules often modulate processes requiring prolonged regulation, such as metabolism, growth, and development.

An example of these processes can be seen in lipolysis, where the body breaks down fats to generate lipids that are subsequently used as precursors in various biological processes. One example where lipids serve as precursors involves the synthesis and secretion of eicosanoids, a family of hormone-like lipids, such as PGs. PGs are derived from arachidonic acid, a polyunsaturated fatty acid released from the phospholipid layer of cell membranes during lipolysis. This fatty acid is then converted into PGE2 through a series of enzymatic reactions involving cyclooxygenase enzymes (COX-1 and COX-2) and PGE synthase [91]. Once produced, PGE2 is secreted from the cell and can bind to its specific receptors. PGs play key roles in the metabolic crosstalk among adipose tissue, the immune system, and the liver. In the context of obesity, PGs released from adipose tissue contribute to inflammation by recruiting immune cells to the adipose tissue, promoting a chronic low-grade inflammatory state often seen in metabolic syndrome. Additionally, PGE2 communicates with the liver to regulate lipid metabolism [92]. This example illustrates how lipolysis can rapidly initiate the production of lipid-derived secreted signaling molecules that can control a wide range of physiological processes.

## Biosynthesis, production, and degradation

### Protein and peptide biosynthesis

**Figure 2:**
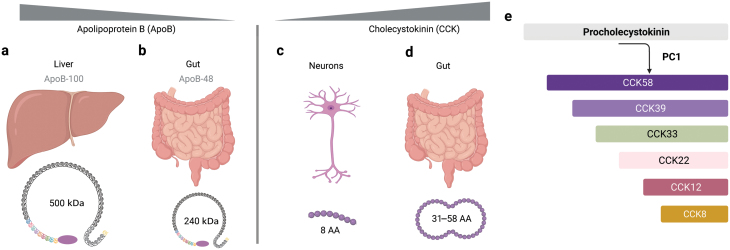
Tissue-specific contrast of size and structure. Evolutionary processes build proteins and synthesize peptides with specific characteristic features fine-tuned to make them bioactive, versatile, and dynamically regulated. (a and b) Splicing of apolipoprotein generates two specific proteins, ApoB-100 of 500 kDa in the liver (a) and ApoB-48 of 240 kDa in the intestine (b). (c and d) CCK, a proteolytically processed peptide hormone composed of eight amino acids, acts as a neurotransmitter in the brain (c). In contrast, in the gastrointestinal tract, the size of CCK varies from 31–58 amino acids and it functions as a digestive peptide hormone (d). (e) The enzyme prohormone convertase 1 (PC1) serially cleaves the prohormone, procholecystokinin (proCCK), into peptides of different size.

Mechanistic understanding of the synthesis of secreted molecules is important to appreciate their regulation and physiological function. Secreted proteins are synthesized based on the genetic information encoded in mRNA. They have an N-terminal signal sequence for secretion following synthesis through translation on ribosomes. Processing of precursor proteins can give rise to distinct peptides with different functions via two mechanisms: mRNA splicing by mRNA editing enzymes, or posttranslational processing by enzymes in the endoplasmic reticulum. Peptide hormones are most often synthesized as prohormones (precursor proteins), which are then cleaved to generate the active hormone [93]. For posttranslational processing, the precursor protein has an N-terminal signal sequence, and the secreted chain is flanked with specific proteolytic enzyme sites. Cell type specific enzyme expression enables the posttranslational generation of fragments of the precursor proteins to generate specific proteins or peptides. Apolipoprotein (Apo)-B100 and Apo-B48 are two distinctive apolipoproteins encoded from the same gene, *APOB* [90]. Apo-B48 is a protein with a molecular weight of 240 kDa (Fig. 2a), and ApoB100 is a very large protein with a molecular weight of > 550 kDa (Fig. 2b). The exclusive presence of the mRNA editing enzyme Apobec-1 in intestinal cells of most vertebrates truncates the *APOB* gene for efficient chylomicron formation and lipid absorption [94]. While both facilitating fatty acid transport by forming lipoprotein complexes, Apo-B100 is exclusively present in the liver and Apo-B48 in the intestine [95]. Cholecystokinin (CCK), a peptide hormone composed of only eight amino acids, acts centrally as a neurotransmitter and regulates food intake (Fig. 2c). In contrast, in the gastrointestinal tract, the size of CCK is larger around 31−58 amino acids, and it acts as a peptide hormone involved in digestion (Fig. 2d) [70, 71]. In addition, several other CCK peptides have been reported (Fig. 2e).

### Metabolite and lipid biosynthesis

Metabolites and lipids are synthesized through metabolic pathways involving a series of enzymatic reactions. They can be degraded by enzymes or spontaneously decompose. Lactate, once considered to be a waste product and fatigue agent, has proved to be the metabolite phoenix as major energy fuel and primary precursor for gluconeogenesis [96]. While not functioning as an endocrine factor, lactate does bridge the gap between glycolysis and oxidative metabolism and also fuels the oxidative machinery of mitochondria in glycolytically active cells. The lactate shuttle hypothesis considers this interconnection to occur under aerobic conditions within and among cells, tissues, and organs [46]. Examples of this lactate shuttle phenomenon can be seen in the exchange between skeletal muscle and other tissues such as the brain, liver, heart, and kidney. This shuttling is regulated through concentration gradients and is mediated by the monocarboxylate transporter (MCT) family, ensuring that lactate fulfills its role in metabolic coordination without invoking the specialized signaling typically associated with hormones. Beyond its role as an energy substrate, lactate contributes to the biosynthesis of L-lactate-derived amino acids, which could be considered endocrine molecules. Li *et al*. demonstrated that exercise-induced secretion of N-lactoyl-phenylalanine (Lac-Phe) helps in reducing adiposity and body weight by suppressing appetite [50]. Lac-Phe synthesis, a reaction catalyzed by the cytosolic enzyme carnosine dipeptidase 2 (CN2 or CNDP2), involves the condensation of lactate and phenylalanine. Intriguingly, the non-specificity of CNDP2 allows it to catalyze the condensation of different amino acids (such as leucine, isoleucine, and valine), leading to the synthesis of diverse N-lactoyl-amino acids. The utilization of the highly dynamic substrate lactate to generate metabolites not only broadens the responsive elements to physical activity but also contributes to long-lasting endocrine effects. This example of a substrate-driven production of bioactive metabolites represents a large, understudied area where many similar metabolites as expected to exist. Another example is the synthesis of T3 (and thyroxine [T4]). This process is unique because it involves the transformation of a protein, thyroglobulin, into a metabolite hormone [97]. T3 synthesis starts with the iodination of tyrosine residues within the thyroglobulin protein, a step that requires the specialized enzyme thyroperoxidase. The iodinated tyrosine residues within the thyroglobulin molecule are then coupled to form the hormones T3 and T4. The thyroid gland has a remarkable ability to concentrate iodine, which is essential for the iodination of tyrosine residues on thyroglobulin, a precursor protein in the thyroid hormone synthesis process. Few metabolic pathways are directly dependent on a specific dietary mineral. Second, the biologically active form of the hormone, T3, is predominantly formed outside the thyroid gland through a process known as peripheral deiodination. This is an unusual feature in hormone metabolism because the full activation of the hormone occurs not at the site of its synthesis but in the peripheral tissues where it exerts its effect. The flexibility of this process allows the body to fine-tune the amount of active T3 hormone available based on the metabolic demands of the body, further emphasizing the unique nature of thyroid hormone synthesis and regulation. Therefore, large quantities of T3 and T4 can be stored within the thyroid gland as part of the thyroglobulin protein and then released when needed. This unique aspect of thyroid hormone synthesis and storage allows for the rapid release of hormones when the body’s metabolic demands increase [4].

### Metabolites as biomarkers

With the advancements in metabolomics techniques, the identification of small metabolites has exponentially increased and enabled us to discover the novel biomarkers involved in the pathophysiology of metabolic and non-metabolic diseases. Changes and perturbations in the metabolite signature of organisms could help in the advance diagnosis of diseases and drug designing. Molecular metabolite profiling will enable a deeper understanding of the metabolic aspects of diseases and develop early therapeutic interventions [98]. For instance, the galactose/glycerolipid metabolic pathway is disturbed in diabetic kidney disease, suggesting glycerol-3-galactoside as a potential biomarker [99]. Circulating metabolites, hexanoylcarnitine, kynurenine, and tryptophan, have been associated with improvement in the prediction of all-cause mortality in type 2 diabetes [100].

### Degradation

The initiation of a signaling event to facilitate a specific cellular response necessitates the termination of that signal. In circulation, proteins and peptides undergo degradation either extracellularly via proteases or intracellularly through proteases or the ubiquitin-proteasome pathway. Molecules exposed to extracellular spaces are vulnerable to enzymatic degradation, which can impact the signal range [101, 102]. However, stable signal molecules can traverse longer distances. For instance, metabolites like lactate can rapidly diffuse out of the cellular environment. This speed is dependent on their concentration gradient and the availability of transporters. Additionally, the enzymatic conversion of lactate back to pyruvate can occur within seconds to minutes, contingent upon enzymatic activity and substrate availability. Similarly, lipids like PGE2 are rapidly degraded, with a half-life of less than a few minutes in circulation and in tissues [103]. Uptake of PGE2 by cells can occur within seconds, and its enzymatic degradation by 15-hydroxyprostaglandin dehydrogenase (15-PGDH) in the cytoplasm can also occur within seconds to minutes. Peptides such as GLP-1 are degraded and inactivated extremely quickly by dipeptidyl peptidase-4 (DPP-4), resulting in a half-life of only about 1–2 min [104]. This rapid degradation ensures that insulin secretion is tightly regulated and can quickly respond to changes in blood glucose levels. In contrast, proteins like leptin, due to their larger size and complexity, have a slower degradation rate, with a half-life of 40 min in mice [105]. Their degradation, occurring either through proteolysis in the kidneys and liver or via receptor-mediated endocytosis in target cells, can take minutes to hours. This duration depends on factors such as protein stability and the availability of receptors and proteolytic enzymes. These examples underscore the wide range of speeds at which different types of signaling molecules can be degraded, ranging from seconds for small metabolites and lipids to minutes or even hours for larger proteins.

## Evolutionary adaptability of endocrine hormones

### Diversification of signaling molecules and adaptability in evolution

Small molecules could be considered more ancient in the context of cellular signaling and the evolution of life. Before the emergence of complex life forms, primitive cells relied on simple chemical interactions to facilitate cellular processes and communication. Small molecules would have been more accessible and easier for these primitive cells to synthesize and utilize than larger, more complex molecules such as peptides and proteins. As life evolved and organisms became more complex, so did the signaling molecules used for communication between cells and organs. The emergence of peptides and proteins as signaling molecules would have allowed for greater specificity, selectivity, and versatility in cellular communication and regulation of physiological processes. However, small molecules have remained essential components of cellular signaling and metabolic processes in all organisms, from bacteria to humans. They continue to serve as neurotransmitters, hormones, and secondary messengers, playing a critical role in various biological processes such as energy metabolism, cell growth and differentiation, and immune responses.

Diversification of signaling molecules forms the bedrock of biological diversity. The emergence of a diverse range of ligands exerts significant selection pressure on enzymes to evolve [106]. The accumulation of a ligand library, a consequence of evolutionary exposure spanning billions of years, introduces incremental advancements. For instance, gene superfamilies encoding conus peptides rapidly evolve through gene duplications, enzyme mutations, and functional deletions in response to environmental changes [107]. The ability to use a variety of signaling molecules allows for the development of complex, adaptable regulatory systems that can evolve to meet new challenges.

### A great mystery: why are there so many different chemical types of endocrine hormones?

In mammals, secreted signaling molecules are diverse, ranging from small molecules such as metabolites (e.g. catecholamines), lipids and steroids (e.g. PGs and corticosteroids), peptides (2–50 amino acids in size, e.g. GLP-1 and neuropeptides), to larger protein molecules (> 50 amino acids in size, e.g. leptin, insulin, and other tyrosine kinase receptor ligands). Why has nature evolved such chemical diversity in endocrine hormones, and what functional relevance does that have to their signaling? One might speculate that the size, structure, and properties of these molecules have been shaped by evolution to facilitate nuanced control over countless biological processes. The evolution from simpler life forms to multicellular organisms has led to increased diversity in signaling molecules and complexity in signal transduction pathways, driving a dynamic process of intercellular communication. Metabolites, due to their small size and direct dependence on substrate availability, can serve as quick response messengers to changes in cellular metabolic status, such as during exercise [50, 108, 109] or fasting [110]. Lipids and steroids, due to their lipophilic nature, can pass through cell membranes and exert potent modulatory effects on intracellular targets such as nuclear receptors [27]. Peptides, falling between small molecules and proteins in size, not only often act as rapid local regulators (e.g. neuropeptides) but also serve as important hormonal regulators (e.g. incretins) [111, 112]. Finally, proteins, the largest and most diverse group of signaling molecules, can exert a wide array of effects on cells due to their structural and functional diversity [33, 113, 114]. This diversity ensures that the endocrine system can fine-tune its responses according to the distinct needs of different physiological states.

### Evolutionary contrast in insulin size: comparing humans and cone snails

**Figure 3:**
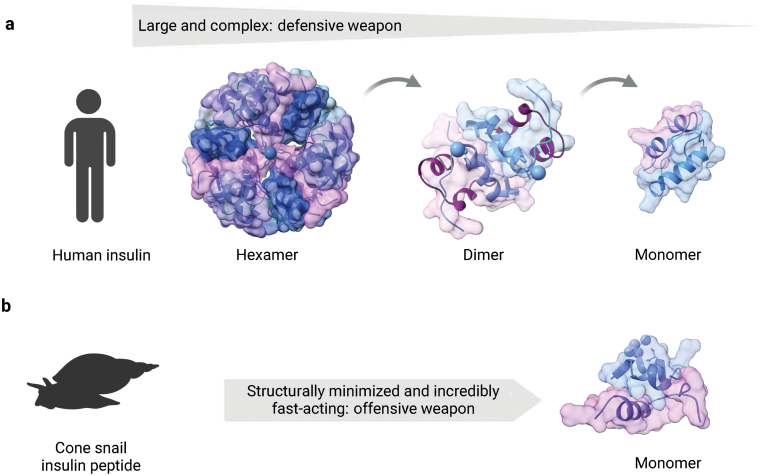
Insulin size architecture tailored by nature. Human insulin has acquired a propensity to self-assemble into dimers and hexamers, protecting itself from enzymatic degradation. To facilitate the glucose uptake of cells and lower circulating blood glucose levels, human insulin hexamer molecules have to first dissociate into dimers and functional monomers (a). In contrast, in cone snails, nature has designed insulin molecules with minimum structure and yet it is fully functional and incredibly fast-acting. The cone snail insulin is readily secreted as a monomeric unit and, in contrast to human insulin, is used as an offensive weapon (b).

The discovery of insulin, a critical hormone for blood glucose regulation, is a milestone in the advancement of drug discovery and enhancing the longevity of humans [2]. Insulin varies remarkably between humans and other species, including, drosophila [115], and cone snails [116]. In humans, insulin is a 51 amino acid (5.7 kDa) protein hormone secreted by pancreatic β-cells and stored in secretory vesicles as hexamers (Fig. 3a). The formation of the hexamer starts with the oligomerization of three dimers held together by zinc ions. Storing insulin as hexamers in secretory vesicles is critical as it protects insulin from degradation and fibrillation [117]. To facilitate glucose uptake into cells and lower systemic blood glucose levels, the insulin hexamer molecules must dissociate into dimers, and finally into the functional monomer. This insulin monomer not only acts as a signaling ligand for insulin receptors but also has faster pharmacokinetics, resulting in lower stability in circulation. The hexamer-to-monomer transition is a slow process, and this predicament has spurred the engineering of a fast-acting monomer insulin analog for diabetes [118]. The C-terminus of B chain in the insulin molecule facilitates the dimerization process and also confers receptor activation [119]. Intriguingly, the engineering of a fast-acting insulin analog has been done by nature thousands of years ago. In contrast to human insulin, insulin present in the venom of the cone snail, *Conus geographus*, is comparatively a smaller peptide of 10–40 amino acids and is typically monomeric in structure. Cone snail insulin also lacks the C-terminus region responsible for dimerization in human insulin. Interestingly, cone snail insulin is extremely potent and fast-acting in lowering blood glucose in vertebrates, including humans [107]. Cone snails use insulin in their venom as an offensive weapon to catch the prey by causing hypoglycemia that sedates the prey (Fig. 3b) [116]. It is fascinating that to target the different insulin receptors, different fish species fine-tune the ligand specificity by producing different versions of insulin (cone snail insulins (Con-Ins) G1 and Con-Ins G3) (Fig. 3). The rationale for such divergence is the evolutionary pressures stemming from constantly changing prey-predator relationships. Cone snail insulin peptides and other constituents of their venom are the most rapidly diverging and evolving ligands in biology [116]. However, the prey of cone snails is constrained to evolve their insulin receptor specificity. So the evolutionary contrast of insulin in humans and cone snails exemplifies the essence of ligand size in nature. In this example, insulin is united by function and divided by structure and action—used as an offensive weapon in cone snails and defensive in humans.

## Future perspectives

As we continue to deconstruct the biology of cellular communication, we acknowledge the presence of an extensive array of distinct signaling molecules, each possessing unique characteristics and functions. These entities, defined by their individual structure, size, and physicochemical properties, not only carry out designated roles but also contribute to the multidimensional architecture of intercellular and intracellular signaling pathways. Despite the considerable advancements in our understanding of these processes, it is apparent that we are only beginning to explore this vast landscape of signaling biochemistry. The acceleration in the discovery of signaling molecules, propelled by advancements in high-resolution mass spectrometry methodologies, mandates the development of an inclusive and evolving classification system. This system should move beyond the traditional organ-specific classification and include the synthesis, regulation, specificity, and signaling responses of these molecules, thus providing a more integrative view of their physiological implications. As advancements in detection methodologies continue to evolve, we anticipate the identification of an even more diverse collection of these molecules, further enriching our understanding of the intricate landscape of cellular signaling pathways and mechanisms. High-throughput metabolomics combined with deep mining of data from biobanks, such as the UKBioBank, and where feasible, the use of genetic models will be crucial for the discovery and elucidation of the roles of these signaling molecules within a spectrum of physiological and pathological conditions, and for confirming their therapeutic potential. Therefore, continuous endeavor to identify novel signaling molecules and subsequent elucidation of their physiological roles will remain a central theme in metabolism in the coming years.
